# Postpartum resolution of hypertension, proteinuria and acute kidney injury among women with preeclampsia and severe features at Mulago National Referral Hospital, Uganda: a cohort study

**DOI:** 10.4314/ahs.v23i3.6

**Published:** 2023-09

**Authors:** Kasereka Muteke, Milton W Musaba, David Mukunya, Jolly Beyeza, Julius N Wandabwa, Paul Kiondo

**Affiliations:** 1 Department of Obstetrics and gynecology, College of Health Sciences, Makerere University, P.O BOX 7072, Kampala, Uganda; 2 Department of Obstetrics and Gynecology, Faculty of Health Sciences, Busitema University, P.O Box 1460, Mbale, Uganda; 3 Department of Public and Community Health, Faculty of Health Sciences, Busitema University, P.O Box 1460, Mbale, Uganda; 4 Department of Obstetrics and Gynecology, Mulago National Referral Hospital, P.O Box 7051, Kampala, Uganda

**Keywords:** Acute kidney injury, preeclampsia with severe features

## Abstract

**Background:**

The resolution of hypertension, proteinuria and AKI postpartum among women with preeclampsia is not well documented in Uganda.

**Objective:**

To determine the time to resolution of hypertension, proteinuria and AKI postpartum until 6 weeks among women with preeclampsia in Mulago Hospital, Uganda.

**Methods:**

Between August 2017 and April 2018, we measured blood pressure, urine protein and serum creatinine on days 1,7,21 and 42 postpartum among 86 women with preeclampsia. The primary outcomes were time to the resolution of hypertension, proteinuria and AKI. We fitted accelerated failure models using Stata 17's *stintreg*. command with a log normal distribution and obtained time ratios of selected exposures on time to resolution of hypertension, proteinuria and AKI intervals.

**Results:**

The median time to resolution of hypertension, proteinuria and AKI was seven (7) days (Inter quartile range, IQR 1-21). The time to resolution of hypertension among primiparous women was 3.5 times that of multiparous women [TR 3.5, 95%CI 1.1, 11.3]. No differences were observed in resolution of hypertension, proteinuria and acute kidney injury.

**Conclusion:**

The time to resolution of hypertension, proteinuria and AKI was seven days. We recommend larger studies with longer follow-up beyond six-weeks postpartum to inform revision of our guidelines.

## Introduction

Hypertensive disorders of pregnancy complicate approximately 10% of all pregnancies worldwide^1^. Preeclampsia or eclampsia are a form of hypertensive disorders of pregnancy associated with proteinuria or end-organ dysfunction or both, is associated with high maternal and perinatal morbidity and mortality 2, 3. In 2017, almost all 277,300/303,000 (94%) of the annual maternal deaths occurred in low- and middle-income countries. Sub-Saharan Africa alone accounted for nearly two-thirds (196 000) of these maternal deaths, while Southern Asia accounted for nearly one-fifth (58 000)^4^, ^5^. At 1 in 59 pregnancies, sub-Saharan Africa has the highest lifetime risk of maternal death, compared to 1 in 160 pregnancies for the rest of the low-income countries^6^ and 1in 5,400 pregnancies for high income countries^7^. Furthermore, at 27 deaths per 1,000 live births, sub-Saharan Africa has the highest neonatal mortality in the world^8^. Majority of the maternal and perinatal morbidity and mortality occurs in the intrapartum and immediate postpartum period^4^, ^5^ ^9^ In Uganda, 11% of the 1,083 maternal deaths audited and reported to the ministry health in the year 2019 to 2020 were caused by preeclampsia or eclampsia^10^. Worldwide, preeclampsia or eclampsia is the second leading cause of direct maternal deaths^11^. In the year 2019 to 2020, preeclampsia or eclampsia was the third leading cause of maternal deaths after postpartum hemorrhage (46%) and sepsis (13%) in Uganda^10^. In Mulago National Referral Hospital, the largest maternity hospital in Uganda, preeclampsia is the leading cause of maternal deaths and it contributes to 8% of the severe maternal morbidity^12^.

Renal impairment, especially acute kidney injury (AKI) is a common complication of preeclampsia or eclampsia^13^. It is present in 15% of all patients with preeclampsia or eclampsia and it is associated with a 50% increased risk of maternal and fetal death^11^. Reported estimates of perinatal mortality among patients with pregnancy related acute kidney injury range from 23.8% to 38^14^. On the other hand, a systematic review and meta-analysis review reported 13.3% maternal deaths among women with acute kidney injury due to various pregnancy related causes including preeclampsia^14^. Bentata et al. reported a higher maternal mortality of 28.3% among pregnant women with AKI admitted to their ICU^15^, which is not surprising because generally, patients with similar conditions admitted to ICU tend to have higher death rates compared their counterparts not admitted to the ICU.

Pregnancy related acute kidney injury is more common in low- and middle-income countries and its reported incidence is 4-26% compared to 1-2.8% in high-income countries^14^. Yet little is known about the epidemiology of acute kidney injury especially among women with preeclampsia or eclampsia in low- and middle-income countries. In most women, hypertension, proteinuria and AKI resolve after childbirth^16^, ^17^, but for some, these complications may persist or even become permanent sequalae. Evidence from epidemiological studies has shown that preeclampsia is associated with increased risk of development of cardiovascular and renal diseases such as of end-stage renal disease in future when patients are followed for as long as 17 years^18^. For instance, in the CRADLE trial, more than two thirds of women with preeclampsia or eclampsia fully recovered from the pregnancy related acute kidney injury at the end of the follow up period of six weeks^19^. The authors were not in a position to give any clear indication of the rate of development of CKD in patients with AKI due to preeclampsia because the follow-up was short, and many patients were lost to follow-up 20. In some patients with preeclampsia, it may take up to two years for hypertension and proteinuria to resolve 16. For that matter, Conti-Ramsden et al^19^ advise that investigations for chronic hypertension and CKD should be deferred until after 2 years.

In low- and middle-income countries, the prevalence, risk factors and complications associated with preeclampsia and eclampsia have been well documented 21-27. Knowledge of the rate of resolution of hypertension, proteinuria, and acute kidney injury among women with preeclampsia with severe features will inform the formulation of guidelines for closer monitoring and management of these patients. Such women may require longer follow up and investigations to identify those at risk of developing chronic hypertension or renal dysfunction 16, 28. An earlier study from the same hospital evaluated the incidence and factors associated with persistent hypertension in women admitted with pre-eclampsia/eclampsia^22^, it did not look at the resolution of proteinuria and acute kidney injury. In this study, we evaluated the resolution of hypertension, proteinuria and AKI postpartum until 6 weeks among women with preeclampsia with severe features in Mulago Hospital.

## Materials and Methods

### Study design

This was a prospective cohort study conducted from August 2017 to April 2018 in Uganda. This study was approved by the Mulago hospital ethics committee, the Makerere School of Medicine Research and Ethics committee and Uganda National Council for Science and Technology. Written informed consent was obtained from the participants.

### Study setting

This study was conducted at Mulago hospital. Mulago hospital is a national referral hospital for Uganda that serves as the teaching hospital for Makerere University College of Health Sciences. Mulago hospital delivers about 30,000 women per year and offers antenatal and postnatal services.

### Study population

The study population consisted of women who had preeclampsia and delivered at Mulago hospital during the study period. Women with a known history of hypertension, diabetes mellitus and kidney disease were excluded from the study.

Preeclampsia was defined according to the classification by the working group of national high blood pressure education program 29(2000) and the American College of Obstetricians and Gynecologists 30(2019). Under this classification, hypertension was defined as a systolic blood pressure of ≥ 140mmHg and/or diastolic blood pressure of ≥ 90mmHg on two occasions at least 4 hours or more apart. Hypertension was also confirmed in a short time or a few minutes when a systolic BP of ≥160mmHg or diastolic BP of ≥110mmHg was recorded. Proteinuria was defined as urine protein of ≥300mg/24h urine collection or protein/creatinine ratio of ≥ 0.3 or a dipstick reading of ≥2+. Preeclampsia was taken as hypertension with proteinuria after 20 weeks of gestation. In absence of proteinuria, preeclampsia was taken as hypertension with one or more severe features of preeclampsia. Preeclampsia with severe features is defined as any one or more of: severe hypertension ( systolic BP of ≥160 mmHg or diastolic of ≥110 mmHg), headache that is not responsive to simple analgesics, visual disturbances, thrombocytopenia of ≤100,000/µL, aspartate transaminase or alanine transaminase > 2times the upper limit with severe epigastric or upper quadrant pain, pulmonary edema, renal dysfunction (serum creatinine >77 µmol/L), and a convulsion/fit that could not be attributed to any other cause^19^,^31^, ^32^.

We used serum creatinine levels of >77 µmol/L as the cut-off for the definition of AKI 33. We chose this definition over other well-known consensus definitions for acute kidney injury such as kidney disease: Improving Global Outcomes (KDIGO)^34^-^36^, because of the inability to collect a 24-hour urine sample for measurement of proteins, so we only used urine dipstick.

### Sample size calculation

Using the formula described by Kelsey et al in OpenEpi, Version 3, open source calculator^37^, we assumed that the persistence of hypertension would be 42.6% as was found in a study by Kaze et.al^38^ and parity as a biggest risk factor for preeclampsia with an odds ratio of 3.71 as was found in a study in Mulago hospital 27. With these estimates a sample size of 82 participants would be sufficient with power of 80% at confidence level of 95% taking in account of the anticipated loss to follow up of 5%.

### Study procedures

The research assistants who were qualified midwives identified women with preeclampsia with severe features from the labour ward and the high dependence unit of the hospital. They approached the women and gave them information about the study and obtained written informed consent. The eligible participants were recruited consecutively until the required sample size was achieved. The information from the women was collected using an interviewer-administered questionnaire, participants' examination, and biochemical investigations. Urine was collected from the women for urine protein estimation and blood was drawn for serum creatinine measurement.

### Follow up

The women were followed for 6 weeks after delivery. The women were reviewed on days 1,7,21 and day 42 by the research team. During the review the women were asked about their health and a focused history and examination were done using case record form. The blood pressure was measured, blood was drawn from the women for measurement of serum creatinine and urine for estimation of urine protein.

### Outcomes

The primary outcomes were time to resolution of hypertension, proteinuria and AKI up to 42 days of follow up. The blood pressure was considered normal when it was less than 140/90 mmHg without any antihypertensive medications for at least one week.

### Data analysis

Data were analysed using Stata version 17.0 (StataCorp; College Station, TX, USA). Continuous variables were summarized into means, median, and standard deviation. Categorical variables were summarized into proportions.

### Resolution of hypertension

We defined resolution of hypertension as the first visit at which a participant had systolic blood pressure less than 140 mm/Hg and diastolic blood pressure less than 90 mm/Hg. Since resolution obviously occurred between visits, the actual time of resolution was analysed as interval censored. We fitted accelerated failure models using Stata 17's *stintreg*. command with a log normal distribution and obtained time ratios of selected exposures on time to resolution of hypertension. Participants who were lost to follow up and participants in whom no resolution was observed by the last visit (day 42), were considered right censored.

### Resolution of proteinuria

We defined resolution of proteinuria as the first visit a participant had no protein detected by urine dipstick. Since resolution obviously occurred between visits, the actual time of resolution was analysed as interval censored. We fit accelerated failure models using Stata 17's *stintreg*. command with a log normal distribution and obtained time ratios of selected exposures on time to resolution of proteinuria. Participants who were lost to follow up and participants in whom no resolution was observed by the last visit (day 42), were considered right censored.

### Resolution of acute kidney injury

We defined resolution of kidney injury as the first visit a participant had a serum creatinine level less than or equal to 77 mol/L. Since resolution obviously occurred between visits, the actual time of resolution was analysed as interval censored. We fit accelerated failure models using Stata 17's *stintreg*. command with a log normal distribution and obtained time ratios of selected exposures on time to resolution of acute kidney injury. Participants who were lost to follow up and participants in whom no resolution was observed by the last visit (day 42), were considered right censored.

## Results

During the study period, there were 97 women diagnosed with preeclampsia. Of these, two died in the hospital before childbirth and nine did not give informed consent to participate in the study. We recruited 86 women with preeclampsia, of which 54 (62.8%) had acute kidney injury (AKI)

### Participant characteristics

The median systolic blood pressure (± interquartile range 169 (155-180mmHg) and diastolic blood pressure 113 (106 -121). Most of the participants were multiparous 51 (60%) and of these 11 (13%) were grand multiparous. The details are in table 1

**Table 1:** Baseline characteristics of the study participants with preeclampsia

Variable, medians (IQR)	Total
Age, years	27 (23-30)
Weight, Kg	73 (66-82)
Height, Cm	160.2 (157-164)
Systolic blood pressure, mmHg	169.5 (155-180)
Diastolic blood pressure, mmHg	113 (106-121)
Gestation age, weeks	37.5 (34-38)
Early onset ≤ 34 weeks	22 (25.6)
Late onset ≥35 weeks	64 (74.4)
Inter pregnancy interval, years (n=47)	2 (1-3)
Baseline creatinine	90 (72-124)
Baseline urine protein on dipstick (n=73)	4 (3-4)
**Parity**	
Primigravida	34 (40)
G2 to 4	40 (47)
G 5+	11 (13)
History of kidney disease	
Yes	0 (0)
No	86 (100)
History of hypertension	
Yes	0 (0)
No	86 (100)
History of preeclampsia/eclampsia	
Yes	17 (19.8)
No	69 (80.2)

* Only 46 observations. We shall use creatinine clearance too

### Resolution of hypertension after childbirth

**Figure 1:**
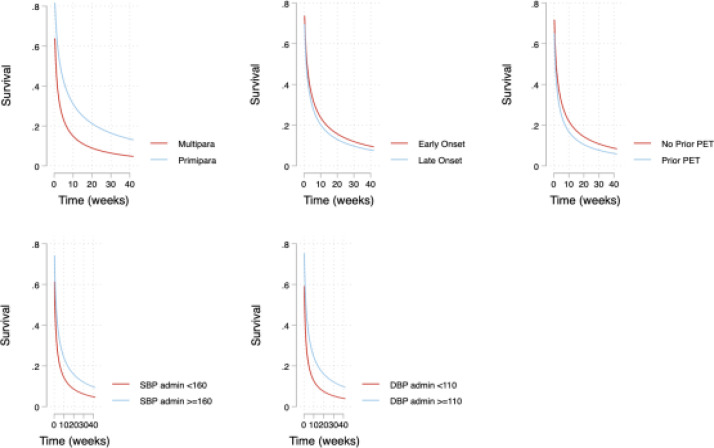
Association between time to resolution of hypertension and selected factors among women admitted with preeclampsia in Mulago hospital

The median time to resolution of hypertension was seven (7) days (Inter quartile range, IQR 1-21). At enrolment 85/86 (98.8%) of the women had hypertension, at delivery 78/86(90.7%) of them had hypertension, on the first day postpartum 51/86(59.3%) had hypertension, at seven days 55/86 (64.0%) were still hypertensive, at 21 days 23/73(31.5%) were hypertensive and at 42 days postpartum 5/73 (6.9%) were hypertensive. Details are in figure 1. time to resolution of hypertension among primiparous women was 3.5 times that of multiparous women [TR 3.5, 95%CI 1.1, 11.3]. No differences were observed in resolution of hypertension between early onset and late onset preeclampsia, those with prior history of preeclampsia and those without, and those with and without severe hypertension at diagnosis. The details are in table 2.

**Table 2:** Time ratios of the accelerated failure time models

Variable	Resolution of HTN	Resolution of protein	Resolution of AKI
	Time ratios (95% CI)		
**Parity**			
Multipara	1	1	1
Primipara	**3.5 (1.1-11.3)**	1.10 (0.77-1.7)	1.10 (0.42-2.7)
**Gestation age**			
Early onset < 34 weeks	1	1	1
Late onset ≥ 34 weeks	0.75 (0.16-3.5)	0.80 (0.48-1.3)	0.64 (0.21-2.0)
**Prior history of preeclampsia**			
No	1	1	1
Yes	0.64 (0.15-2.8)	1.20 (0.73-1.9)	0.88 (0.29-2.7)
**Systolic BP, mm Hg**			
< 160	1	1	1
≥160	2.3 (0.63-8.6)	0.92 (0.60-1.4)	0.84 (0.31-2.3)
**Diastolic BP, mm Hg**			
< 110	1	1	1
≥110	2.8 (0.76-10.5)	0.83 (0.53-1.3)	1.00 (0.37-2.9)

CI, confidence interval.

### Resolution of proteinuria after childbirth;

**Figure 2:**
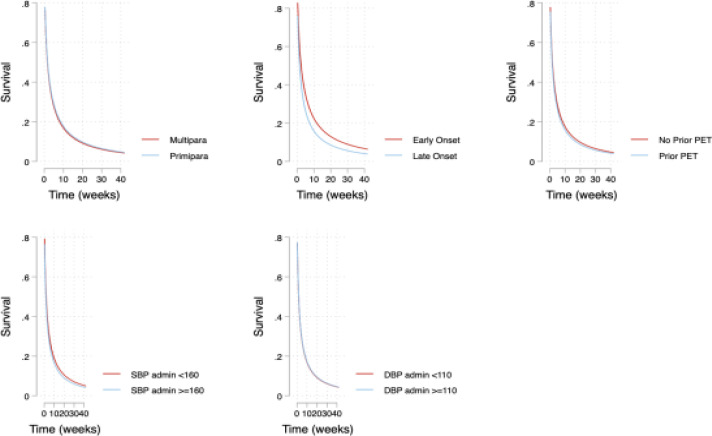
Association between time to resolution of proteinuria and selected factors among women admitted with preeclampsia in Mulago hospital

The median time to resolution of proteinuria on urine dipstick was seven (7) days (Inter quartile range, IQR 1-21). At enrolment 71/86 (82.6%) of the women had proteinuria, on the first day postpartum 51/86(59.3%) had proteinuria, at seven days 46/86 (88.4%) had proteinuria, at 21 days 37/86(43%) had proteinuria and at 42 days postpartum 13/86 (15.1%) still had proteinuria on dipstick. No differences were observed in resolution of proteinuria on the basis of these known risk factors. The details are in table 2 and figure 2.

### Resolution of acute kidney injury after childbirth

**Figure 3:**
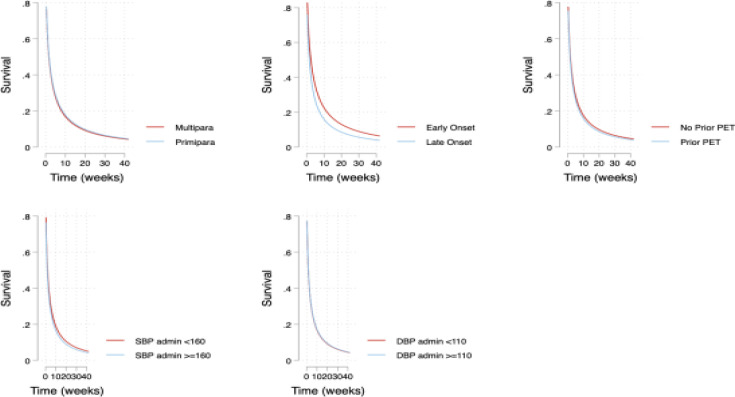
Association between time to resolution of acute kidney injury and selected factors among women admitted with preeclampsia in Mulago hospital

The median time to resolution of AKI was seven (7) days (Inter quartile range, IQR 1-21). At enrolment 54/86(62.8%) of the women had AKI, on the first day postpartum 41/86(47.7%) had AKI, at seven days 39/86(45.4%) had AKI, at 21 days 37/86(39.5%) had AKI and at 42 days postpartum 33/86(38.4%) still had AKI. No differences were observed in resolution of acute kidney injury on the basis of these known risk factors. The details are in table 2 and figure 3.

## Discussion

This was a prospective cohort study in which 86 women with preeclampsia with severe features were followed up to 42 days postpartum. We found that the median time to resolution of hypertension, proteinuria and acute kidney injury was seven days. The time to resolution of hypertension among primiparous women was 3.5 times that of multiparous women. No differences were observed in resolution of hypertension, proteinuria and acute kidney injury on the basis of these known risk factors.

In this study, most women had an earlier recovery than the women in the studies by Wei et al and Mikami et al 39, 40. This was probably because most mothers in our study had late onset preeclampsia at a mean (±standard deviation) gestational age of 35.9±4.0 weeks. Many studies have shown an association between late onset of preeclampsia and early resolution of hypertension^21^, ^38^, ^41^. Furthermore, we found that the time to resolution of hypertension among primiparous women was 3.5 times that of multiparous women. This is not surprising because low parity is a well-known risk factor for preeclampsia with severe features, just like gestation age.

This information is of importance to clinicians in settings where the utilization of postnatal services is very low. It means that this category of patients needs more counselling than is usually offered after childbirth, so that they can adhere to postpartum checks up to the end of puerperium because they are at a higher risk of developing preeclampsia in subsequent pregnancies, and also developing chronic hypertension.

In this study, three quarters of the women had acute kidney injury at enrolment, which was much higher than the 35.3% reported by Prakash in India among women in late pregnancy and the 25% reported by Kaze among women with preeclampsia with severe features and eclampsia in Cameroon 38, 41. The high prevalence in this study was surprising. One possible explanation is that since the study was conducted in a national referral hospital, the patients were very sick and needed to be nursed in high dependency units or intensive care units that were not readily available in lower health units. In addition, these patients might have had some other underlying conditions that affect kidney function that we did no exclude at enrolment such as sepsis^42^, ^43^. The assessment of kidney function in this study was based on the serum creatine level because of challenges in obtaining a 24-hour urine sample, which was a major limitation of the current study. As a marker of kidney injury, serum creatine has several limitations which limit its usefulness^44^, ^45^ ^26^,^27^. Its production, secretion and elimination is influenced by several factors including hemodynamic changes without structural kidney injuries^44^. The current study did not measure early and more specific urinary biomarkers of acute kidney injury such as albumin^44^.This is probably because acute kidney injury is temporary and completely reversible after childbirth^19^, ^38^.

### Methodological considerations

Our study has a larger sample size compared to previous studies conducted in similar settings on the same subject. The main limitation of this study was the inability to collect a 24-hour sample of urine to determine urine protein, this meant we couldn't use the well-known consensus definitions such as KDIGO criteria for acute kidney injury.

## Conclusion

The median time to resolution of hypertension, proteinuria, and acute kidney injury was seven days. We recommend further studies with larger sample size and longer term follow up beyond six weeks to inform the revision of our guidelines.

## Data Availability

The data used to support the findings of this study are available from the corresponding author upon request.
